# The Pathophysiology of Sinking Flap Syndrome Associated with Low-Pressure Hydrocephalus: A Case Study Suggests a New Hypothesis

**DOI:** 10.3390/jcm15124753

**Published:** 2026-06-18

**Authors:** Grant A. Bateman, Alexander R. Bateman

**Affiliations:** 1 Department of Medical Imaging, John Hunter Hospital, Newcastle, NSW 2310, Australia; 2 School of Medicine and Public Health, College of Health, Medicine and Wellbeing, University of Newcastle, Callaghan, NSW 2308, Australia; 3 Discipline of Aerospace, Mechanical and Mechatronics Engineering, School of Engineering, College of Engineering, Science and Environment, University of Newcastle, Callaghan, NSW 2308, Australia; alex.bateman@newcastle.edu.au; 4 Brain Health Research Program, Hunter Medical Research Institute, Newcastle, NSW 2310, Australia

**Keywords:** case report, cerebral blood flow, blood–brain barrier, low-pressure hydrocephalus, sinking flap syndrome

## Abstract

**Introduction:** Decompressive craniectomy (DC) is often required to stabilize the intracranial pressure (ICP) in patients with traumatic brain injury (TBI). Both sinking flap syndrome (SFS) and hydrocephalus are known complications of DC. The pathophysiology of each is unknown. **Case Report:** We report on a patient who underwent DC for TBI who suffered both SFS and low-pressure hydrocephalus. We measured the changes in volumes of each hemisphere and the ventricles with CT and the cerebral blood flow (CBF) and aqueduct flow with phase-contrast MRI during different stages of the disease process. **Discussion:** The SFS in this patient was associated with a reduction in volume of both supratentorial cavities. There was a significant reduction in CBF bilaterally, which increased by an average of 26% following cranioplasty. During the low-pressure hydrocephalus phase of the patient’s illness, there was reversed CSF flow directed toward the ventricles. Once the ventricles returned to normal size, this reversed flow was lost. **Conclusions:** Lumped parameter modelling of the patients’ CSF and vascular systems suggested a new hypothesis, i.e., that the reduction in blood flow was due to reversible constriction of the arterioles secondary to a reset of the autoregulation rather than compression of the venous structures. We suggest there is an increase in CSF absorption efficiency despite the known CSF-absorption mechanisms being unlikely to function at such a low ICP. A hypothesis is put forward that CSF absorption occurs via the brain capillary bed in these diseases.

## 1. Introduction

Decompressive craniectomy (DC) involves the removal of a large portion of the skull to accommodate the increased brain volume, which can occur in patients with severe cerebral edema following a brain insult [1]. Decompressive craniectomy is often required to urgently treat intracranial hypertension due to stroke, head trauma, and cerebritis [2]. A cranial window allows the swollen brain to bulge out through the defect, decreasing the intracranial pressure (ICP) and increasing the intracranial compliance [2]. Usually, the swelling subsides over several weeks, and a bone flap is replaced in the cranial window. The procedure is not without risk, with many possible complications. Of interest is the development of sinking flap syndrome (SFS). In this syndrome, the previously convex skin flap becomes concave and bulges inward toward the brain. The collapse of the flap occurs weeks to months following DC and is associated with headaches, dizziness, seizures, sensory deficits, motor dysfunction, and cognitive impairment [3]. The symptoms improve following reconstitution of the skull with cranioplasty. The incidence is approximately 28% of craniectomies [4]. Another significant complication of DC is hydrocephalus. Hydrocephalus refers to the abnormal accumulation of CSF within the ventricles not due to atrophy or ex-vacuo dilatation [5]. The ICP is often elevated in hydrocephalus but may be in the normal range in so-called “normal pressure hydrocephalus” (NPH) [6]. Rather paradoxically, Pang and Altschuler identified a variant of hydrocephalus with a low or even negative ICP [7]. Decompressive craniectomy leads to hydrocephalus in 15% of patients with traumatic brain injury (TBI) [8]. The purpose of the current paper is to describe the CT and MRI findings in a patient who underwent DC for a TBI who subsequently suffered both sinking flap syndrome and low-pressure hydrocephalus. The aim is to see if this case can provide any clues as to the underlying pathophysiology of these complications and suggest a new hypothesis regarding causation.

## 2. Case Report

A man in his fifties suffered an electric scooter accident while not wearing a helmet. On arrival at the hospital, he was unconscious with a Glasgow Coma Scale (GCS) score of 7. He was noted to have a large abrasion to the right side of his head. He had no movement of his left upper limb and was very weak in the left lower limb, with normal movement on the right. An immediate head CT (Somatom Force, Siemens, Erlangen, Germany) scan showed a large right frontoparietal intraparenchymal hemorrhage (see Figure 1A).

The volume of the hemorrhage was measured by tracing the outline of the hemorrhage on the individual slices and multiplying the areas obtained by the slice thickness. A volume of 94 mL was calculated. A slice obtained at the level of the frontal horns of the ventricles showed a midline shift and some ventricular effacement (Figure 1B). The changes in the volume of the right and left supratentorial spaces, incorporating the midline shift, the ventricular volume, and the total supratentorial volume, were measured using the same technique as for the hemorrhage. The findings are appended in Table 1.

The supratentorial space had a volume of 1300 mL initially, and, given that the right and left hemispheres are usually symmetrical, this would give a volume of 650 mL for each. It is noted that the midline shift had increased the volume of the right side by 32 mL and reduced the left by the same amount. The ventricles were slit-like with a small total volume of 4 mL.

The patient was immediately taken to the operating theater, and a large decompressive craniectomy and hemorrhage evacuation performed. A CT was performed the next day (Figure 1C). The craniectomy had increased the supratentorial volume by 144 mL. there was a minimal reduction in the midline shift, but the ventricles returned to a normal size. A lumbar drain was inserted to monitor and treat the raised ICP. At this point, the GCS improved to between 14 and 15 with good verbal responses, but with a dense left hemiplegia and a left-sided neglect. Over the course of time, the patient developed fluctuating reductions in the level of consciousness and confusion. The lumbar drain was removed on day 18 post-injury.

By day 78 the patient was noted as becoming drowsier, with worsening speech and slurring of his words initially but ultimately becoming almost mute. He developed severe paroxysmal reductions in consciousness, and a repeat CT was performed (Figure 1D). The flap was now deeply concave with 19.2 mm of a midline shift. The sulci appeared compressed, but paradoxically, the left ventricle had continued to increase in size. The diagnosis of sinking flap syndrome was made. The volume measurements showed that the total supratentorial volume had reduced by 156 mL below the initial volume, with 96 mL of this reduction coming from the right side and 60 mL from the left. The ventricular volume had increased by 108% compared to the craniectomy phase. It was feared that the patient was developing obstructed hydrocephalus secondary to kinking of the cerebral aqueduct, so an MRI study with phase-contrast volume measurements of the cerebral blood flow and aqueduct flow (Magneton Vida, Siemens, Erlangen, Germany) was performed on day 81. The results are appended in Table 2.

The right, left, and basilar artery, blood flows were measured at the skull base, and the total flow was calculated by adding these together. The total flow seemed low at 534 mL/min. The aqueduct was patent with no pulsation to the flow. It was noted there was a measured net caudocranial flow of 5 mL/min into the ventricles. The cross-sectional area of the sagittal sinus was measured on the T2 images 3 cm above the torcula. On day 114, a cranioplasty was performed using a polyetheretherketone prosthesis. The patient almost immediately improved with the GCS increasing from 14 to 15. The patient’s speech returned to the initial findings, and he was able to sit up and feed himself. The supratentorial volume returned to just above normal with less midline shift (Figure 1E). However, the ventricles remained enlarged. A repeat MRI study 20 days after the cranioplasty showed an increase in the right carotid flow of 39% and the left 26%. The total blood flow increased by 26%. The aqueduct flow had returned to being pulsatile with a systolic stroke volume of 79 µL. The net CSF inflow towards the ventricles had reduced to 1.5 mL/min. The cross-sectional area of the sagittal sinus was reduced by 26%.

By day 23 following the cranioplasty, it was noted there was a hemo-serous discharge from the cranioplasty wound. Wound swabs performed at this time showed profuse methicillin-susceptible staphylococcus aureus. The patient became febrile, and the wound broke down at the cranioplasty site. The prosthesis was on show and was noted to be surrounded by purulent material on a follow-up CT (not illustrated here). The cranioplasty was removed 3 days later. There was a period of wound infection, which was treated with antibiotics. By day 28 following the removal of the cranioplasty, the sinking flap syndrome had recurred with fluctuating consciousness and reduced speech. A repeat CT (Figure 1F) showed an increased midline shift, but the ventricles were no longer dilated, with no evidence of hydrocephalus. The supratentorial volume was now 59 mL less than the initial volume with a 39 mL reduction on the right and 20 mL on the left. The MRI study was repeated on day 30 following the cranioplasty removal, and the cerebral blood flow was now less than that during the previous sinking flap syndrome episode, being 502 mL/min in total. The aqueduct flow was again non-pulsatile, but there was no net flow into or out of the ventricles at this time. The sagittal sinus had increased in cross-sectional area by 15%. A timeline has been provided for clarification; see Figure 2. A CARE case report guideline check list is provided in Supplementary File S1.

## 3. Discussion

### 3.1. Sinking Flap Syndrome: Brain Compression or Reduced CSF Volume

As noted in the introduction, this patient fulfilled the criteria for sinking flap syndrome (SFS) on two occasions. In addition, the marked dilatation of the ventricles despite the reduced subarachnoid-space CSF volume in Figure 1D suggested the development of low-pressure hydrocephalus, which subsequently resolved over time. The Monro–Kellie doctrine states that as all the components of the intracranial cavity are incompressible within the fixed volume of the skull, and any increase in one component must be accommodated by an equal reduction in another. Thus, the brain, CSF, and cerebral blood volumes must be in balance [9]. The initial hemorrhage acutely added 94 mL of volume to the right hemisphere. This was accommodated by shifting the midline, allowing the right hemisphere to increase in volume by 32 mL. The remaining 62 mL appeared to be accommodated by reducing both the CSF volume within the subarachnoid space on the right and the size of the ventricles. In the acute phase, the brain parenchymal volume would not alter. The cerebral blood flow reduces on average by 23% in TBI in the acute setting, but the CBV does not change significantly [10]. This means that the CSF volume must change acutely. The 32 mL reduction in the left supratentorial space was accommodated by reducing the subarachnoid space over the left hemisphere. Thus, we can see that the increase in volume on the right, which occurred after the initial hemorrhage, pushed the midline of the brain to the left and compressed the CSF spaces bilaterally to dissipate any remaining pressure gradients. Following the craniectomy and the evacuation of the hemorrhage, the brain herniated into the skull defect.

During the first SFS phase (Figure 1D), there was a return of the midline shift (greater than previous) and a reduction in the CSF spaces around the brain to accommodate the overall 156 mL reduction in intracranial volume. As the brain is very compliant, the initial pressure gradient that would have produced these changes would be largely dissipated at this stage [11]. No long-term pressure differences are seen between the ventricles, cortical subarachnoid spaces, and brain parenchyma in either animal models or humans [11]. The time taken for the total pressure to equilibrate within the brain is as short as 35 ms [12]. Hladky and Barrand state that the CSF column pressure is an accurate measure of the total brain pressure in most instances [11]. This raises the question, was the brain pushed (due to an elevated atmospheric pressure gradient) or pulled (due to marked CSF absorption)? It is generally assumed that the brain is pushed by the atmospheric pressure collapsing the flap [3]. Often authors note that the normal air pressure at sea level is 760 mmHg and suggest that a large pressure gradient occurs across the cranial defect to be transmitted through to the entire cerebrum [13]. However, this is incorrect. The air pressure is 760 mmHg with respect to the vacuum of space or compared to the vacuum above the mercury column within a barometer but not with respect to the pressure within the cranium. In patients undergoing decompressive craniectomy, the ICP measured with the frontal lobe with a telemetric sensor in the supine position averaged −0.5 mmHg the week before cranioplasty and increased to 6.3 mmHg in the week following cranioplasty. The sensor depth is between 2.5 and 3 cm in the frontal region [14]. Given that there is a hydrostatic pressure gradient within the brain due to the height of the skull, the average intracranial pressure would be higher than that measured in the frontal lobes. In the case of our patient, if a sensor had been placed at 3 cm, this would be 5.5 cm above the external auditory meatus. If this offset was similar in all patients, then the ICP would be 3.5 mmHg in the cohort with a frontal sensor as discussed. In 11 patients with sinking flap syndrome, the ICP at lumbar puncture averaged between 1.5 and 3.7 mmHg [15]. Finally, Fodstadt et al. found the initial ICP to be 8.3 mmHg in their cohort [16]. Thus, three studies suggest that the average ICP in SFS is above atmospheric pressure. We can estimate the ICP in our patient by looking at the skin flap inflection point (see Figure 3). In this figure, the flap is noted to be sigmoid with both concave and convex components (Figure 3A). The inflection point defines the change from concave to convex (yellow arrow). The ICP is below atmospheric pressure above the inflection point and above atmospheric pressure below this point for this bowing to occur. Thus, at the inflection point, the ICP equals atmospheric pressure. In Figure 3B, we note that the external auditory meatus is approximately 5 cm vertically below the inflection point; thus, the pressure within the CSF column in this patient can be calculated as 3.7 mmHg. As discussed above, the parenchymal pressure is almost always identical to the CSF column pressure, or else the brain moves. Thus, in Figure 1D, the 19.2 mm midline shift is occurring despite the ICP likely being above the atmospheric pressure, and therefore, the brain was unlikely to have been pushed. This indicates that the brain was likely to have been pulled due to a primary reduction in the CSF volume.

There is further evidence that the brain was not pushed. This comes from looking at the size of the sulci on the opposite vertex to the side of the disturbance. This will be explained with the help of Figure 4.

It appears to be very difficult to shift the brain towards the opposite vertex even with a large increase in the volume on the other side because the falx cerebri is firmly attached to the skull. Thus, initially, in Figure 4A, there is obliteration of the sulci on the right but no compression on the left. The left sulci do not change following craniectomy (Figure 4B), confirming they were not originally compressed. In Figure 4C, there is a large reduction in CSF volume on the left despite the brain volume loss on the right due to gliosis from the previous insult. This suggests that the CSF volume on the left is reduced during the SFS despite the left brain not being compressed from the right. This indicates that the CSF volume loss is not due to compression from the outside. The volume of CSF returns to normal following cranioplasty (Figure 4D). Marasanov et al. came to the same conclusion. In a patient with the SFS, CSF volumes were measured with MRI volumetry. Pre-cranioplasty, the cranial CSF volume was 100.8 mL and the spinal volume was 78.2 mL. These volumes increased to 152.7 mL (+51.9 mL) and 91.1 mL (+12.9 mL), respectively, following cranioplasty with no change in the brain parenchymal volume [17]. Indicating that the CSF volume is the primary driver of the SFS, with the reduced volume even occurring within the spinal canal. It is known that volume loss and crowding of the sulci over the vertex, such as that seen in Figure 4C, is a hallmark of NPH, further suggesting that our patient initially had both sinking flap syndrome and hydrocephalus at the same time [18]. Given that a suggested threshold for the classification of low-pressure hydrocephalus is an ICP less than 70 mmH_2_O or 5.1 mmHg [19], our estimate of the average ICP in this patient being 3.7 mmHg indicates that this is likely to be low-pressure hydrocephalus rather than NPH.

### 3.2. Changes in Cerebral Blood Flow

Our findings of a total CBF of 530 mL/min for the first instance of sinking flap syndrome and 502 mL/min in the second are much less than that expected for a male in his mid-50s, which is usually closer to 600 mL/min [20,21]. In a wide-ranging review of the CBF changes in DC patients following cranioplasty, Halani et al. found that all studies demonstrated an increase in the ipsilateral CBF, with 43% of studies reporting increased flow on the contralateral side as well [13]. In a study of 54 patients measuring the effect of cranioplasty on CBF using first-pass perfusion CT, the mean increase in CBF and cerebral blood volume (CBV) on the craniectomy side was 15% and 8%, respectively. On the contralateral side to the skull defect, the CBF and CBV increased by 14% and 12%, respectively [22]. However, individual case reports can show remarkably large improvements in CBF and CBV up to 220% and 27%, respectively, on the ipsilateral side and 270% and 26% on the contralateral side [23]. Therefore, our findings of a 39% and 26% increase in the ipsilateral and contralateral CBF are not without precedent. However, we are unaware of any other studies using phase-contrast MRI to measure this effect. As to why such a reduction in blood flow occurs, the literature brings up similar suggestions as noted before. Halani et al. speculate that the external force transmitted from the atmosphere to the cerebral vasculature may be enough to cause reduced CBF due to incomplete vessel collapse [13]. However, is an average pressure within the intracranial cavity of 3.7 mmHg, as in our patient, enough to collapse the vessels? To investigate this further, we have performed a lumped parameter study using a technique that we have previously extensively published [24,25,26,27,28] (see Figure 5). This model has been validated by correctly predicting the CBV in many differing disease states given the changes in the CBF and ICP. The current modeling is based on the current patients’ CBF and the mean arterial pressure (which was 100 mmHg at the time of the SFS). The ICP is our estimate of an average of 3.7 mmHg in this patient, and the venous sinus pressure comes from Fodstadt et al., who used CSF infusion studies in 14 patients with sinking flap syndrome following craniectomy and calculated the sagittal sinus pressure to be 5.6 mmHg [16].

Essentially, the modeling balances the changes in blood pressure across each segment with the calculated changes in blood volume for each segment depending on the relationship between the change in volume and the change in resistance. More information can be obtained by reviewing the original papers. The main finding is that the CBF is reduced below a normal middle-aged person despite the perfusion pressure (mean arterial pressure—ICP) being increased from 88.5 to 96.5 mmHg. This means that the total vascular resistance must increase by 44%. The venous sinuses are predicted to dilate due to the reversed pressure gradient across their walls (+1.9 vs. −4.0 mmHg). This correlates well with the measured sagittal sinus cross-sectional area in Table 1 being dilated during the SFS phases compared to the post-cranioplasty state. In spontaneous intracranial hypotension, the sagittal sinus cross-sectional area was 22% larger than in controls due to the reduction in the transmural pressure [29]. The marginal volume decrease in the parenchymal veins comes about due to the slight decrease in the venous transmural pressure gradient, but the increased resistance due to this is negligible. The capillary bed is not compressed in this model due to the extreme bending forces required to reduce such small vessels in volume any further despite the reduced transmural pressures. To balance the total resistance, the arterioles must be significantly constricted, reducing their volume. The total CBV of 47.4 mL in this patient is 7% less than normal and compares with the previously discussed literature findings that the CBV is reduced by an average of 8% in the sinking flap syndrome on the ipsilateral side [22]. The latter prediction suggests the modeling is accurate enough for the current purposes. Note that the arterioles constrict despite having a much larger internal pressure than the ICP, indicating they are not being compressed from outside. The fact that the arterioles are so constricted but can dilate to increase the CBF following cranioplasty indicates they are not irreversibly damaged but rather are electing to be constricted. The same finding was noted in NPH, where the average CBF is reduced by 20% and increases following treatment [26]. This is another indicator that sinking flap syndrome and NPH share a pathophysiology. Changes in the CBF following TBI are common. Depending on the type and magnitude of the injury, a global reduction in blood flow occurs within 12 h of injury, often with reactive hyperemia following this [30]. In the current study, the reduced CBF occurs much later than these acute changes. Vasospasm due to constriction of the blood vessels is common in TBI but is normally resolved by day 14–30 [30]. This also does not fit with our current patient’s time line. More chronic vascular injury may take longer to repair but would not be expected to respond acutely to cranioplasty or to deteriorate again secondary to removal of the prosthesis.

### 3.3. Increased CSF Absorption Efficiency

We have concluded that a reduction in the CSF volume is the primary abnormality in the SFS. This could come about due to a lack of CSF formation or an increase in the efficiency of CSF absorption. Fodstadt et al. used CSF infusion studies on post-craniectomy patients with sinking flap syndrome and found the CSF formation rate to be normal at 0.42 mL/min [16]. This suggests that the CSF absorption must be occurring with an average set point (ICP) of 3.7 mmHg in our patient. This would make the absorption mechanism of increased efficiency. It is difficult to see how the standard CSF outflow pathways could even operate at such low pressure, let alone at increased efficiency. Most of the CSF absorption is thought to occur through the arachnoid villi. The opening pressure for cranial arachnoid villi is around 3–5 mmHg above the sinus pressure [31]. In Figure 5, you can see the driving pressure across the sinus wall is estimated to be −1.9 mmHg, i.e., no flow should occur. Some CSF absorption is hypothesized to occur via the olfactory nerves into the nasal/ cervical lymphatics [32] and also is suggested to occur through the lymphatics adjacent to the sinuses [33]. The lymphatics drain into the central veins in the chest, which sit at a pressure of 5.0 mmHg [34], meaning that the driving pressure for lymphatic absorption is usually 6.5 mmHg between the CSF and right heart. This falls to −1.3 mmHg in our patient, meaning that lymphatic absorption is unlikely to be operating at an increased efficiency. Previously, we have suggested that the brain opens an accessory CSF-absorption pathway via the capillaries in NPH and in idiopathic intracranial hypertension by utilizing the Starling forces across the capillary walls [25]. Net fluid absorption or production follows a relationship depending on the balance of hydrostatic and osmotic pressures and is summarized in Equation (1).

(1) $$J_{cap}=L_{cap}[\left\{P_{cap}-P_{CSF}\right\}-\sigma_{cap}\left\{\pi_{cap}-\pi_{CSF}\right\}]$$

where J_cap_ is the net capillary fluid flow rate, L_cap_ is the capillary hydraulic conductivity coefficient, P_cap_ − P_csf_ is the hydraulic pressure gradient across the capillary wall (i.e., the transmural pressure), σ_cap_ is the osmotic reflection coefficient, and π_cap_ − π_csf_ is the osmotic pressure gradient across the capillary wall taking into account both salt and protein [35]. It was noted previously that with an intact blood–brain barrier, the cerebral capillaries have hydraulic conductivities that are two to three orders of magnitude less than systemic capillaries (effectively zero) [36], and the osmotic reflection coefficient is 1 for all substances [35], meaning that no net production or absorption of water is possible [25]. However, if the blood–brain barrier opens, then the hydraulic conductivity would increase and the reflection coefficient for salt would drop to zero. Under these circumstances, CSF absorption is possible provided that the capillary transmural hydrostatic pressure is low enough and the osmotic pressure gradient afforded by the higher concentration of protein within the capillaries compared to the CSF holds by retaining a relatively high reflection coefficient for protein [25]. We note that the average capillary transmural pressure in Figure 5 is reduced in the SFS by 28% compared to normal. This is principally due to an almost 50% reduction in the average capillary bed pressure. There is a significant blood–brain barrier disruption in both NPH [37] and sinking flap syndrome [38]. In hydrocephalus, the CSF protein concentrations are not altered compared to normal in patients with either communicating high-pressure hydrocephalus or NPH [39]. The total CSF protein was noted to be low before the shunt but increased by 135% one month after the shunt in NPH [40]. The finding of normal or low CSF protein in NPH would tend to suggest some retention of the protein reflection coefficient. Ten patients with sinking flap syndrome following DC had total CSF protein levels measured [3]. Excluding the three patients who had shunts inserted, there was a mean level of 7% above the mean for a large cohort of neurologically normal individuals [41]. This suggests that the reflection coefficient for protein must be near normal in the SFS. We cannot estimate the exact protein reflection coefficient, but we can perform a sensitivity analysis using a range of values from 1 to 0.5. A coefficient much less than 0.5 should result in a significantly increased CSF protein level. Thus, if the hydraulic conductivity coefficient were increased, the reflection coefficient for salt reduced to zero, and the protein reflection coefficient retained between 1 and 0.5 in SFS; then, CSF absorption through the capillaries would be possible. Is this hypothesis feasible? We can test this by placing the known and estimated values in equation 1 to see if an acceptable result occurs. If all of the normal CSF production calculated by Fodstadt et al. in SFS to be 0.42 mL/min was absorbed through the capillaries, then J_cap_ would be −0.42 mL/min. We calculated the P_cap_ − P_CSF_ to be 8.7 mmHg in Figure 5. The π_cap_ − π_CSF_ for protein is 25 mmHg [42]. Our estimate for the reflection coefficient for protein is between 1 and 0.5. Placing these values in equation 1 gives a hydraulic conductivity coefficient between 0.026 and 0.11 mL/min/mmHg, respectively. For a 1500 g brain, this would be between 0.002 and 0.007 mL/min/mmHg/100 g, which is 85–44% lower than the figure for skeletal muscle, which is 0.01–0.015 mL/min/mmHg/100 g [43]. These results suggest that our hypothesis may be feasible.

### 3.4. Low-Pressure Hydrocephalus vs. Sinking Flap Syndrome

Thus, both low-pressure hydrocephalus and SFS require a CSF absorption of greater than normal efficiency, which we hypothesize may be due to capillary absorption. What then is the difference between SFS and hydrocephalus? This patient had two episodes of SFS; in the first, the ventricles dilated, and in the second, they did not. In the first, the measured CSF flow through the aqueduct was very high and reversed; in the second, there was no flow into the ventricles at all (see Figure 6).

A flow of 5 mL/min into the ventricles (Figure 6A) equates to 7 L per day and is almost certainly an overestimate. Due to partial voluming effects (where a pixel encompasses both flowing and non-flowing spins), the phase contrast technique that we used tends to overestimate the flow volume [44]. However, the effect is stable and relatively limited provided that the number of pixels per vessel diameter is greater than 4. The overestimate error is about 10% at 6 pixels per vessel diameter, 15% at 5 pixels per diameter, and 17% at 4 pixels per diameter [44]. Below 4 pixels per diameter, the technique becomes less stable, with overestimation peaking at about 40% at 3 pixels per diameter [44]. The pixel size that we used was 0.63 × 0.63 mm^2^, meaning that the error for the arterial measurements was 10% or less, but the error for the aqueduct measurement approached 40%. However, the same 3T scanner (Magneton Vida, Siemens, Erlangen, Germany) was used for each measurement, so a similar error should have been seen across all the measurements, meaning that the percentage changes should be accurate. We can discount the CSF flow magnitude due to error, but this should not change the direction of the flow, which should also remain accurate. Irrespective, the net flow was into the ventricles with the ventricles being dilated, and there was no flow into the ventricles with normal-sized ventricles. In 16/21 NPH patients, retrograde flow into the ventricles was seen before shunting and reversed to antegrade after [45]. The authors of this paper noted that retrograde flow indicates a positive pressure gradient between the ventricles and parenchyma, dilating the ventricles [45]. Thus, the only difference between low-pressure hydrocephalus and SFS may be where the CSF is absorbed, through the ventricle walls into the deep white matter in hydrocephalus, giving dilatation, and through the leptomeninges into the cortical capillaries, where no ventricular dilatation ensues.

## 4. Conclusions

The sinking flap syndrome in this patient was associated with a reduction in volume of both of the supratentorial cavities. There was a significant reduction in CBF bilaterally, which increased by an average of 26% following cranioplasty. During the low-pressure hydrocephalus phase of the patient’s illness, there was reversed CSF flow directed toward the ventricles. Once the ventricles returned to normal size, this reversed flow was lost. Lumped parameter modeling of the patients’ CSF and vascular systems suggested that the reduction in blood flow was not due to irreversible vessel injury. We suggest that it may be due to a reversible constriction of the arterioles secondary to a reset of the autoregulation rather than compression of the venous structures. There is evidence to suggest that there may be an increase in the CSF-absorption efficiency despite the known CSF-absorption mechanisms being unlikely to function at such a low ICP. We put forward a hypothesis that CSF absorption occurs via the brain capillary bed in these diseases.

## Figures and Tables

**Figure 1:**
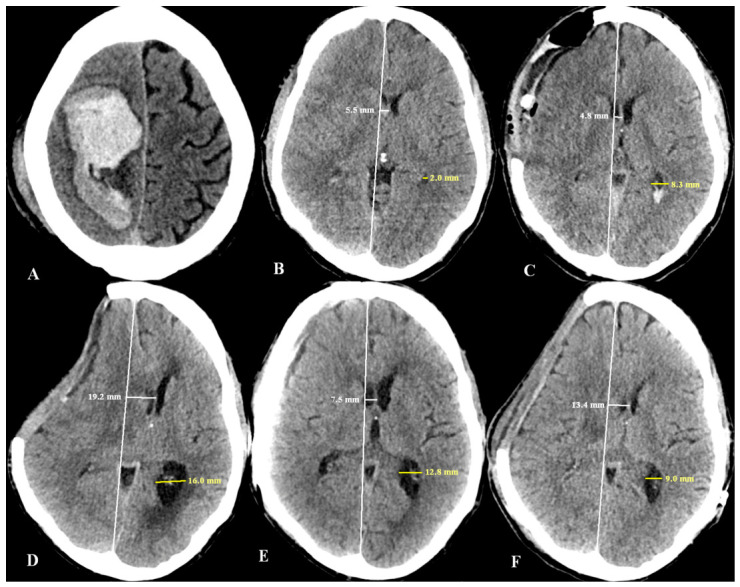
The CT findings during the varying phases of the patient’s hospital stay. (**A**) The initial scan shows a large hemorrhage on the right. (**B**) A lower slice through the brain during the initial scanning showing a 5.5 mm midline shift (white horizontal bar) and a small occipital horn of the ventricle on the left measuring 2 mm (yellow bar). (**C**) The next day following craniectomy, the midline shift had not significantly changed at 4.8 mm. There was bulging of the flap outward. There was swelling of the right hemisphere with loss of the cortical sulci. The left occipital portion of the ventricle had increased in size to 8.3 mm. (**D**) The sinking flap phase was associated with a deeply concave skin flap on the right with 19.2 mm of the midline shift and reduced cortical sulci bilaterally. The occipital portion of the lateral ventricle had dilated to 16 mm. (**E**) Following cranioplasty, the midline shift had reduced to 7.5 mm; the cortical sulci had returned, and the left ventricle had slightly reduced in size to 12.8 mm. (**F**) Following removal of the cranioplasty, the midline shift had increased to 13.4 mm, but the ventricles continued to decrease in size.

**Figure 2:**
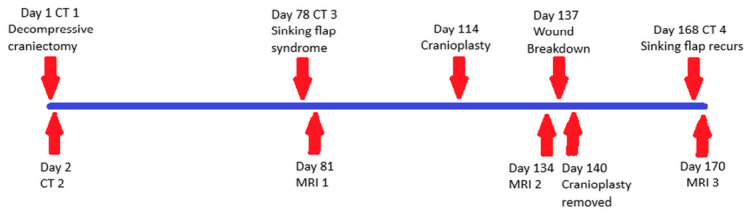
A timeline of the patient’s progress.

**Figure 3:**
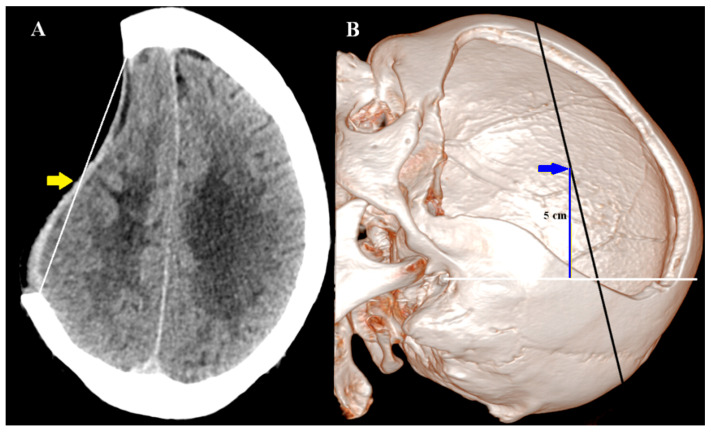
Estimating the ICP from the flap inflection point. (**A**) A CT slice from the center of the flap during the sinking flap phase. The white line shows the skin flap to be sigmoid in shape with concave and convex components. The yellow arrow is the inflection point. (**B**) A 3D reconstruction showing the bony defect with the black line showing the position of the slice from Figure 3A. The arrow is the level of the inflection point. The white line is the level of the external auditory meatus. The inflection point is 5 cm above the external auditory meatus (blue line).

**Figure 4:**
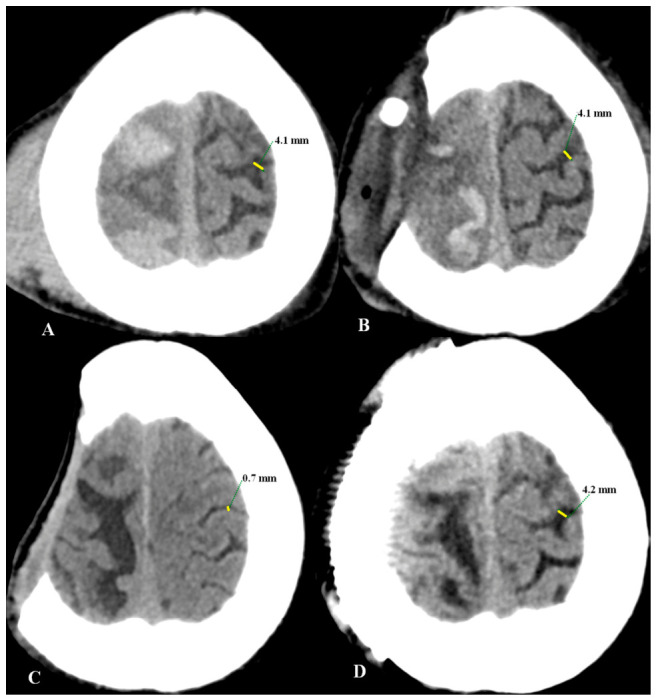
The CT findings at the vertex during the varying phases of the patient’s hospital stay. (**A**) The initial scan shows loss of the sulci on the right but preservation on the left, with the central sulcus measuring 4.1 mm (yellow bar). (**B**) Same slice position after craniectomy showing the left sulci to have not changed in volume. (**C**) During the sinking flap phase, there is a reduction in the size of the sulci of 83%. (**D**) Following cranioplasty, the sulci return to a normal volume.

**Figure 5:**
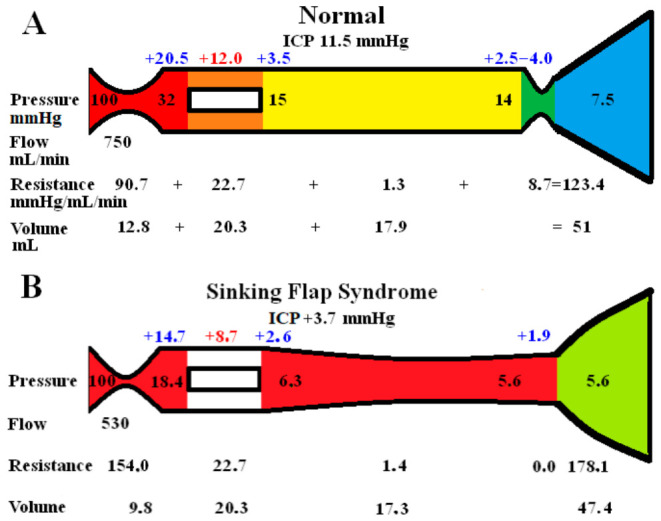
Lumped parameter modeling of sinking flap syndrome compared to normal. The five vascular segments modeled are shown in (**A**): the arteries in red, the capillaries in orange, the veins in yellow, the outflow cuff in green, and the sinus in blue. The pressures at the beginning and end of each segment have been appended within the vessels in (**A**) and were originally obtained from the literature. The resistance of each segment was calculated to be the change in pressure divided by the flow and is shown below the vessels. The normal cerebral blood volume (CBV) values are shown below the resistances. The blue numbers represent the transmural pressure gradients for each vessel segment and are obtained by subtracting the ICP from the segment pressure. The red numbers are the average capillary TMP obtained by averaging the value from before and after the capillaries. (**B**) represents the findings in sinking flap syndrome; the red segments represent the areas of increased resistance compared to the normal findings, and the green represents reduced resistance. Note: ICP, intracranial pressure; min, minute; mm, millimeter; and mmHg, millimeter of mercury. (**A**) is reproduced from [26] under a CC BY 4.0 commons license.

**Figure 6:**
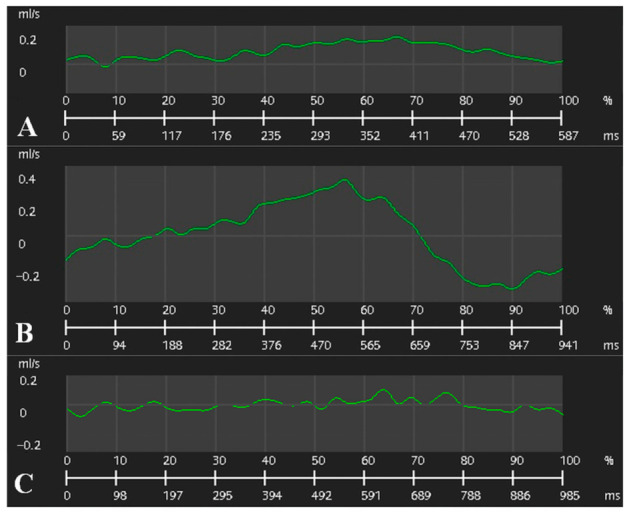
Flow graphs through the aqueduct. (**A**) During the first sinking flap syndrome phase, there was little pulsation, indicating high intracranial compliance. There was no negative flow indicating only flow passing intracranially. (**B**) During the period of the cranioplasty, a normal pulsation returns with a reduction in the net flow, but this still occurs into the ventricles. (**C**) The second sinking flap syndrome period shows no pulsation or net flow into or out of the ventricles.

**Table 1:** Summary of CT volume changes.

Region	Acute	Craniectomy	Sinking Flap 1	Cranioplasty	Sinking Flap 2
Right ST mL	682	807	554	705	611
Left ST mL	618	637	590	648	630
Total ST mL	1300	1444	1144	1353	1241
Ventricular mL	4	13	27	28	10

Note: mL, milliliters; ST, supratentorial.

**Table 2:** Summary of MRI findings.

Measurement	Sinking Flap 1	Cranioplasty	% Change	Sinking Flap 2	% Change
Right carotid flow mL/min	174	242	+39	172	−29
Left carotid flow mL/min	204	258	+26	192	−26
Basilar flow mL/min	156	174	+12	138	−21
Total CBF mL/min	534	674	+26	502	−26
Aqueduct pulsation µL	0	79	-	0	−100
Aqueduct net flow mL/min	5	1.5	−69	0	−100
Sagittal sinus area mm^2^	73.1	54.3	−26	62.4	+15

Note: CBF, cerebral blood flow; µL, microliters; mL/min, milliliters per minute; mm^2^, millimeters squared.

## Data Availability

All data utilized is contained within the article.

## Supplementary Materials

The following supporting information can be downloaded at: https://www.mdpi.com/article/10.3390/jcm15124753/s1, File S1: CARE checklist.

## Abbreviations

The following abbreviations are used in this manuscript:

CBF	cerebral blood flow
CBV	cerebral blood volume
CSF	cerebrospinal fluid
CT	computed tomography
DC	decompressive craniectomy
GCS	Glasgow coma scale
Hg	mercury
ICP	intracranial pressure
Jcap	net capillary fluid flow rate
Lcap	capillary hydraulic conductivity coefficient
min	minute
mL	milliliter
mm	millimeter
MRI	magnetic resonance imaging
NPH	normal pressure hydrocephalus
Pcap	capillary hydrostatic pressure
Pcsf	CSF hydrostatic pressure
SPS	sinking flap syndrome
TBI	traumatic brain injury
µL	microliters
πcap	capillary osmotic pressure
πcsf	CSF osmotic pressure
σcap	capillary reflectance coefficient
