# Supplementary material for: The Pathophysiology of Sinking Flap Syndrome Associated with Low-Pressure Hydrocephalus: A Case Study Suggests a New Hypothesis
Source: J Clin Med. 2026 Jun 18;15(12):4753. doi: 10.3390/jcm15124753 (PMC13301116; doi:10.3390/jcm15124753)
Supplement: Supplementary file 1 [file jcm-15-04753-s001.zip › jcm-4362752-supplementary.pdf]

CARE Checklist of information to include when writing a case report

CARE case report guidelines

(cc) BY-NC-ND

Topic	Item	Checklist item description	Reported on Line
Title	1	The diagnosis or intervention of primary focus followed by the words "case report"	line 5
Key Words	2	2 to 5 key words that identify diagnoses or interventions in this case report, including "case report"	line 37
Abstract (no references)	3a	Introduction: What is unique about this case and what does it add to the scientific literature?	line 20-21
	3b	Main symptoms and/or important clinical findings	line 21-22
	3c	The main diagnoses, therapeutic interventions, and outcomes	line 25-30
	3d	Conclusion—What is the main "take-away" lesson(s) from this case?	line 30-36
Introduction	4	One or two paragraphs summarizing why this case is unique (**may include references**)	line 63-65
Patient Information	5a	De-identified patient specific information.	line 69
	5b	Primary concerns and symptoms of the patient.	line 72-74
	5c	Medical, family, and psycho-social history including relevant genetic information	line 69-160
	5d	Relevant past interventions with outcomes	line 135-138
Clinical Findings	6	Describe significant physical examination (PE) and important clinical findings.	line 71-73,115-117
Timeline	7	Historical and current information from this episode of care organized as a timeline	line 162
Diagnostic Assessment	8a	Diagnostic testing (such as PE, laboratory testing, imaging, surveys).	Line 76,100,129,243
	8b	Diagnostic challenges (such as access to testing, financial, or cultural)	N/A
	8c	Diagnosis (including other diagnoses considered)	line 73,120,124
	8d	Prognosis (such as staging in oncology) where applicable	N/A
Therapeutic Intervention	9a	Types of therapeutic intervention (such as pharmacologic, surgical, preventive, self-care)	line 106-107,136
	9b	Administration of therapeutic intervention (such as dosage, strength, duration)	N/A
	9c	Changes in therapeutic intervention (with rationale)	line 145-150
Follow-up and Outcomes	10a	Clinician and patient-assessed outcomes (if available)	N/A
	10b	Important follow-up diagnostic and other test results	line 151-160
	10c	Intervention adherence and tolerability (How was this assessed?)	N/A
	10d	Adverse and unanticipated events	line 145-150
Discussion	11a	A scientific discussion of the strengths AND limitations associated with this case report	line 273-425
	11b	Discussion of the relevant medical literature **with references**.	line259,273-283,
	11c	The scientific rationale for any conclusions (including assessment of possible causes)	line 360-372
	11d	The primary "take-away" lessons of this case report (without references) in a one paragraph conclusion	line 464-479
Patient Perspective	12	The patient should share their perspective in one to two paragraphs on the treatment(s) they received	N/A
Informed Consent	13	Did the patient give informed consent? Please provide if requested	Yes ☑ No ☐
